# Radar Sensing for Activity Classification in Elderly People Exploiting Micro-Doppler Signatures Using Machine Learning

**DOI:** 10.3390/s21113881

**Published:** 2021-06-04

**Authors:** William Taylor, Kia Dashtipour, Syed Aziz Shah, Amir Hussain, Qammer H. Abbasi, Muhammad A. Imran

**Affiliations:** 1 James Watt School of Engineering, University of Glasgow, Glasgow G12 8QQ, UK; kia.dashtipour@glasgow.ac.uk (K.D.); Qammer.Abbasi@glasgow.ac.uk (Q.H.A.); Muhammad.Imran@glasgow.ac.uk (M.A.I.); 2 Mobile Health, Centre of Intelligent Healthcare, Coventry University, Coventry CV1 5RW, UK; syed.shah@coventry.ac.uk; 3 School of Computing, Edinburgh Napier University, Scotland EH10 5DT, UK; A.Hussain@napier.ac.uk

**Keywords:** activity detection, machine learning, radar sensing, wireless sensing

## Abstract

The health status of an elderly person can be identified by examining the additive effects of aging along with disease linked to it and can lead to ‘unstable incapacity’. This health status is determined by the apparent decline of independence in activities of daily living (ADLs). Detecting ADLs provides possibilities of improving the home life of elderly people as it can be applied to fall detection systems. This paper presents fall detection in elderly people based on radar image classification by examining their daily routine activities, using radar data that were previously collected for 99 volunteers. Machine learning techniques are used classify six human activities, namely walking, sitting, standing, picking up objects, drinking water and fall events. Different machine learning algorithms, such as random forest, K-nearest neighbours, support vector machine, long short-term memory, bi-directional long short-term memory and convolutional neural networks, were used for data classification. To obtain optimum results, we applied data processing techniques, such as principal component analysis and data augmentation, to the available radar images. The aim of this paper is to improve upon the results achieved using a publicly available dataset to further improve upon research of fall detection systems. It was found out that the best results were obtained using the CNN algorithm with principal component analysis and data augmentation together to obtain a result of 95.30% accuracy. The results also demonstrated that principal component analysis was most beneficial when the training data were expanded by augmentation of the available data. The results of our proposed approach, in comparison to the state of the art, have shown the highest accuracy.

## 1. Introduction

The growing numbers of elderly people present numerous challenges within the healthcare industry. These new challenges demand new methods which can be implemented in a cost-effective manner [1]. Assistive technology can serve to improve the care of elderly people. The healthcare industry is looking to technology to support growing needs, as demonstrated in [2,3,4]. Advances in sensor technology are currently being used to support the elderly population [5] and can be used to detect large-scale body movements such as walking, sitting down on a chair, standing up from a chair, picking up objects, fall events, etc.

The World Health Organization (WHO) has reported that 42% of people over 70 years old experience fall at least once a year [6], which can be caused by various hazards such as slippery floors and low lighting conditions, and the damage can be greater due to elderly people’s bodies being fragile [7]. The risk of falling also increases with age. Falls in elderly people can cause more serious injuries than in younger people [8]. Conditions such as arthritis and visual impairment can increase the chance that an elderly person will fall [9]. Arthritis is a condition that causes pain in the joints [10]. This pain when moving could potentially result in elderly people falling as they struggle to move. Visual impairment can cause the elderly to struggle to see hazards and thus trip and fall. Conditions such as nocturia, which causes older people to wake up during the night with a need to go to the toilet, can pose a greater risk of falling as they try to manoeuvre around the home when tired and under poor lighting [8]. Falling can risk serious injury which can result in hospital visits or permanent disabilities [11]. This can lead to sufferers of falls to no longer be able to live independently for fear of further injury. Moreover, fall events are the second leading cause of accidental death [12].

In this context, various sensing technologies, such as wearable accelerometer devices, vibration sensing, visual sensing and radar-based sensing [13], have been used to monitor daily routine activities of elderly people. Wearable devices use accelerometers to be able to detect the movement of the person wearing the device and several factors must be considered, as elderly people may not wear the devices if they find them uncomfortable [14]. Additionally, elderly people may suffer from conditions that inhibit their memory. They may forget to wear the device at times, and it will no longer be able to sense the movements of that person. Vibration, visual sensing and radar-based sensing work without having any devices introduced to the body. These methods are classified as non-invasive sensing methods. Vibration sensing works using a microphone to record the vibrations on the floor as a person experiences a fall [15]. Visual sensing can be achieved by leveraging camera-based technology. Cameras can be used to record elderly people and frames can be analysed using machine learning techniques to assess if a fall has occurred. It also allows for caregivers to view the situation and decide if further care is required [16]. Cameras may not be ideal as some elderly people will not agree to be under constant surveillance. Radar-based sensing works by exploiting the Doppler signatures created on radar when a fall occurs [17]. This paper aims to implement different machine learning and data processing techniques to improve the existing results of an available dataset containing radar micro-Doppler spectrograms of human activities. The research detailed in this study focuses on the effectiveness of radar-based sensing in detecting activities of daily living. Machine learning (ML) algorithms are applied to a dataset to predict if these activities can be classified using artificial intelligence (AI). The machine learning algorithms used are random forest (RF), K-nearest neighbours (KNN), support vector machine (SVM), long short-term memory (LSTM), bi-directional Long short-term memory (BiLSTM) and convolutional neural network (CNN).

The paper is organised into the following sections. Section 2 provides detailed related work within the field of radar-based motion detection. Section 3 presents the methodology of how the experimentation of this research has been achieved. Section 4 discusses the results obtained through the experimentation. Section 5 gives the conclusion.

## 2. Related work

The following section provides details with regard to state-of-the-art work in the field of human activity recognition using radar sensing technology in conjunction with machine learning methods that have been applied for classification purposes. The work of [18] used the same dataset used in this paper. The paper used SVM, KNN and GoogleNet algorithms to classify the data. The results produced using these algorithms had 78.25% accuracy for SVM, 77.15% accuracy for KNN and 74.70% accuracy for GoogleNet. The work of our paper seeks to improve on these results. The paper [19] included radar spectrograms of activities such as walking, falling, sitting down and bending down. The spectrograms were used for image classification and images were converted to greyscale for data pre-processing. The algorithms deep neural network (DNN) and SVM were applied to the pre-processed data. DNN achieved accuracy results of 87.00% while SVM achieved an accuracy score of 78.00%. Research in [20] used spectrograms for image classification using greyscale. The images were then classified using the SVM algorithm. The authors developed sequential forward selection (SFS) for feature selection. The results of the classification achieved between 92.00% and 95.00% accuracy depending on the number of features used. The paper [21] included the algorithms stacked recurrent neural network (RNN) with LSTM and deep CNN to classify six human motions using radar Doppler images. The six motions were boxing, hand clapping, hand waving, piaffe, jogging and walking. The results achieved in these experiments showed that the stacked recurrent neural network (RNN) with LSTM achieved 92.65% accuracy, with the deep CNN achieving 82.33% accuracy. Erol and Amin [22] used spectrograms and range maps of human motion and fused them together for classification. The spectrogram results are similar to the experiments conducted in our study. The study investigated five human motions, namely bending, falling, sitting, kneeling and walking. The spectogram experiment used obtained a result of 82.24% accuracy using KNN with PCA. With fusion methods of the spectrograms and range maps, we were able to achieve a result of 93.94% using KNN with PCA. The work of [23] used impulse-radio ultra-wideband (IR-UWB) radar to capture 12 kinds of motions. The data processing used KNN to define the features in the spectograms. Then, the power spectrum and Doppler shifts were extracted and sent to a CNN algorithm for classification. This was run using five-fold cross validation and achieved up to 98% for detecting the human motions. The study [24] used IR-UWB radar with CNN to be able to identify between falling and activities of daily living. This work used binary classification and focused on differentiating between the falling motion and any other type of activity around the home. The CNN algorithm was able to provide an accuracy score of 96.35%. The authors of [25] made use of UWB radars to create a dataset that contained 10 subjects aged between 22 and 39 years old, performing 15 different activities. The data were collected while other people were still active in the building behind walls. This was to simulate a realistic care home environment where other residents reside in neighbouring apartments. The experiment achieved an accuracy score of 80% using the random forest algorithm. The work of [26] used a UWB radar with seven subjects carrying out four activities, namely walking, sitting, standing and simulated falling. The collected data were run using 10-fold cross validation and it was found that KNN performed the best with a result of 94.90% accuracy. The authors of [27] used UWB radars to collect data for binary classification of falling and non-falling events. Data were collected using 10 volunteers in three different locations within a proposed apartment. They validated their results by using the leave one subject out method and found that the achieved accuracy was 90.00% using a CNN–LSTM architecture deep learning model.

## 3. Methodology

In this section, we discuss our proposed methodology for fall detection in elderly people. This study makes use of radar micro-Doppler spectrograms of human activities. These data were taken from [18] and are publicly available The data were collected for 99 elderly people at nine different locations, namely the University of Glasgow, a north Glasgow elderly care home and Age UK West Cumbria. The dataset can be found at http://researchdata.gla.ac.uk/848/, accessed on 20 April 2020. The dataset consists of six motions: walking, sitting down, standing up, picking up an object, drinking water and falling. The dataset of the radar spectrograms is in the format of 227 by 227-pixel PNG image files. There is a total of 1633 spectrograms in the dataset. Table 1 shows the breakdown of how many spectrograms are present for each motion.

**Figure 1:**
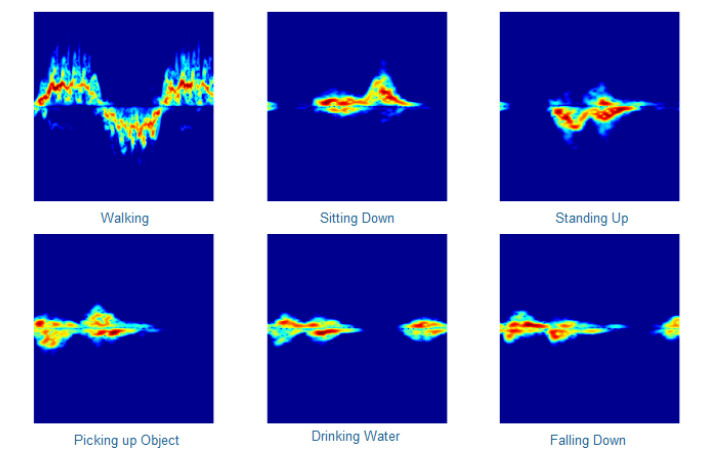
Raw spectrogram examples of each activity [18].

**Figure 2:**
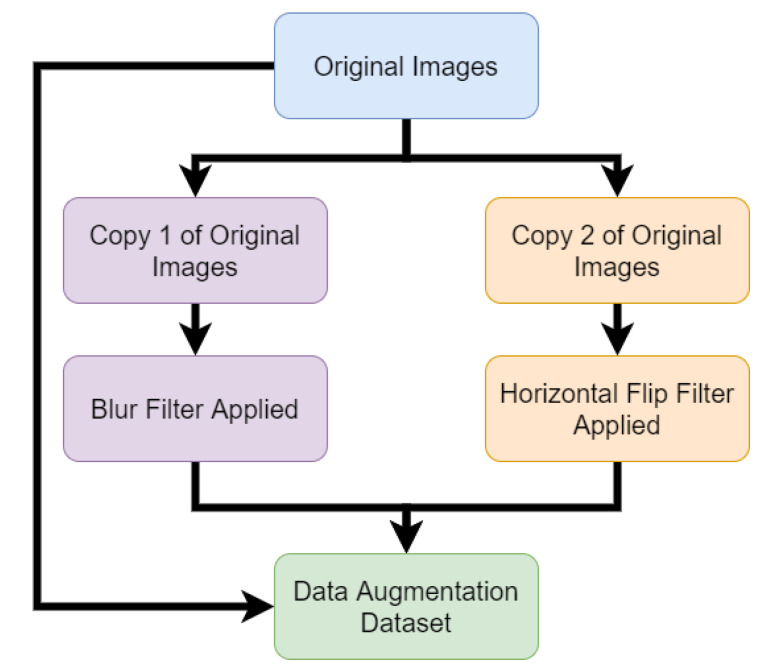
Flow graph of how data augmentation dataset was created.

Figure 1 shows spectrogram examples of each activity. The Skimage Python package was used to convert the image files into data based on pixel information and store this data in Python arrays [28]. The image data array was in the shape of [227,227,3]. This represents the 227-pixel width and 227-pixel height of the PNG file. The 3 represents the red, green and blue (RGB) values of the pixels. The images were then converted into greyscale which removed the colour and changed the array shape to [227,277,1]. This array of data was saved in a CSV format for machine learning processing. The conversion to greyscale was used to reduce the dimensions. The RGB channels were thus converted to a single intensity value [29]. Greyscale images have been shown to have improved classification accuracy over coloured images [30] and this method has been used in various related works involving radar image classification [20,31]. The experiments conducted in this study used four different data processing methods. The first method used the raw greyscale pixel data only. The second method then applied principal component analysis (PCA) to the raw greyscale pixel data. PCA is a technique used to reduce dataset dimensions [32]. PCA finds common components of the data which can summarise the variation of the data [33]. The third method used data augmentation of the raw greyscale pixel data. Data augmentation is the process of increasing the training data by transforming an existing sample [34]. The original and transformed samples can then be part of the same dataset and thus the amount of training data is increased. The transformed samples must keep the variance intact that defines original sample labels within the dataset. PCA is applied in this study by using sklearn. Sklearn provides a function that can fit and transform the image data using PCA. PCA is configured to use all components of the data. Data augmentation in our study tripled the size of the dataset. The data augmentations contain the original greyscale images, blurred greyscale images and horizontally flipped greyscale images. Using blurred and flipped images increases the size of the dataset but these occurrences are unlikely to happen in real life systems. The reason this was carried out in this work is that it allows for more data to be produced while still maintaining some of the original features of the data without causing duplicates in the dataset. The fourth and final method used data augmentation and PCA combined. Figure 2 shows the process followed when creating the data augmentation dataset. Using these four data processing techniques, four different datasets were produced that could then have machine learning and applied and results analysed. The datasets were divided into 90% for training data and 10% for testing data. This was achieved by using the train_test_split function included in the sklearn python package. The function parameters were used to define the percentage of testing data and the rest was used as training data. The data were shuffled but the random seed parameter was always the same to ensure all algorithms used the same samples as testing and training data. This allowed for the results to reflect the model generalisation of the algorithms due to the specific training data remaining consistent and ensuring that the testing samples were the same unseen samples in every experiment conducted.

### Machine Learning

We have used different machine learning algorithm, such as random forest, KNN, SVM, LTSM, BiLTSM and CNN. The Scikit python package was used to implement random forest, KNN and SVM. Scikit is widely used in industry and research within the field of data science [35]. To implement the neural network algorithms LTSM, BiLTSM and CNN, the Tensorflow Python package was used. Tensorflow was used for this research as it is open source and can be used with a graphics processing unit (GPU) [36]. The use of a GPU allows for neural network processes to run faster than if the processes were to be run on a CPU.

The random forest algorithm is a collection of decision trees. Each individual decision tree will make a decision on which classification to assign to new data based on key features learnt in the training phase. The classification each tree reaches is considered a vote. The final classification decided by the random forest algorithm is the ensemble classification of the combined decision trees [37]. In these experiments, the random forest was configured to use 200 trees in the forest. This number of trees resulted in the best results in the initial experimentation.

The KNN algorithm simply looks to compare training data to new unseen data [38]. The features of the training data are assigned K values. The features of the new data are then assigned a K value which best matches the features of the training data [39]. The parameter used for KNN in this study was set to use 10 neighbours.

SVM aims to create boundaries between each classification. These boundaries are known as hyper planes. The hyper plane is positioned as far as possible from the closest data points of the classes present in the data. These points are known as the support vectors [40]. The hyper planes are used to divide the support vectors into the different categories. The training data features are used to place the hyper planes. Then, the features of the new data are used to place the new data between the hyper planes and provide classification [41]. The SVM parameters included the gamma set to auto and the use of the RBF kernel in these experiments.

**Figure 3:**
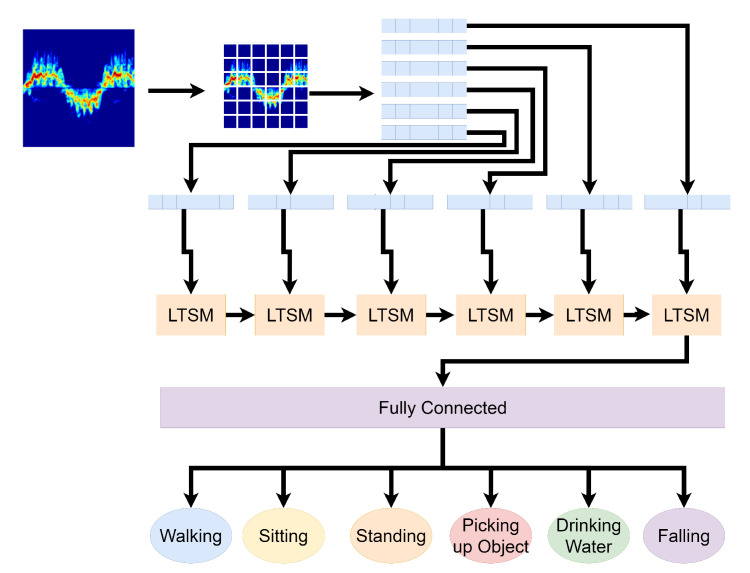
Visual representation of how LSTM works.

The LSTM deep learning algorithm is an extension of a recurring neural network (RNN). A recurring neural network is a type of neural network which models the dynamic behaviour of sequences of data between nodes of the neural network. LSTM expands on this concept by adding three different gates to the nodes. One gate is used to decide if the current state should be forgotten. Another gate is used to control if input should be read or not and the final gate decides if the state should be added to the node output. These gates allow for LSTM to decide if the sequence of data is relevant to the output of the node [42]. In this experiment, LSTM was used with 50 nodes, also known as units. The learning rate was set to 1 × 10^−6^ using ReLU activation and ran for 200 epochs. Figure 3 shows a visual representation of how LSTM works.

**Figure 4:**
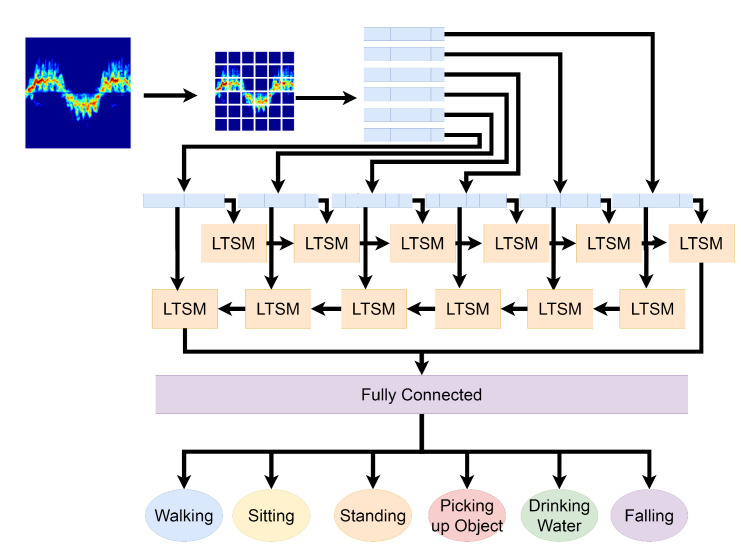
Visual representation of how BiLSTM works.

The bi-directional long short-term algorithm is an extension of the LSTM deep learning algorithm. Where LSTM only makes use of the past behaviours of data sequences, the bi-directional long short-term algorithm can look at data in two directions. The two directions look at past and future data in a sequence. This is achieved by using two LSTM networks. One LSTM network, the forward LSTM network, can review past data sequences and the backward LSTM network can review future data sequences [43]. BiLSTM was configured to use the same parameters as the LSTM set up. Figure 4 shows a visual representation of how BiLSTM works.

**Figure 5:**
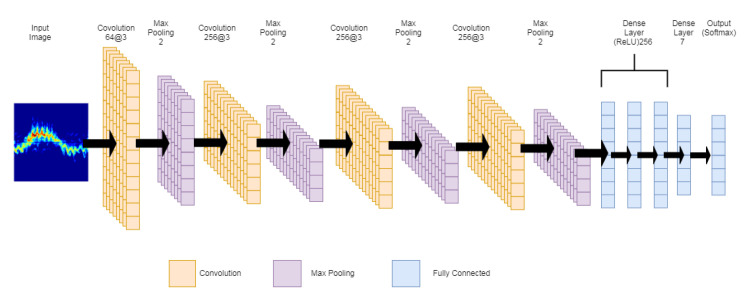
Visual representation of CNN model used in experiment.

The CNN algorithm is an emerging technology which is a powerful solution for image classification problems which were initially thought to require human intelligence [44,45]. The CNN algorithm is made up of densely connected layers that take the activations of all the previous layers as input. The layers produce feature maps from this input which are known as growth rates [46]. The convolution neural network architecture used in this study consisted of multiple convolutions and pooling layers followed by multiple connected layers (ReLU) and finally a softmax layer for creating the output classes. The convolution and pooling layers were used to capture the features of the images. The weight and bias of the features were parameters of the CNN which were configured automatically during the training process [47]. Figure 5 displays the CNN model used in this experiment.

## 4. Results and Discussion

This section presents the weighted average results of each of the classifications carried out in each experiment. Each experiment includes the weighted average accuracy in bar chart format and then the weighted average accuracy, precision, recall and F1-score.

### 4.1. Original Dataset

#### 4.1.1. Raw Images

**Figure 6:**
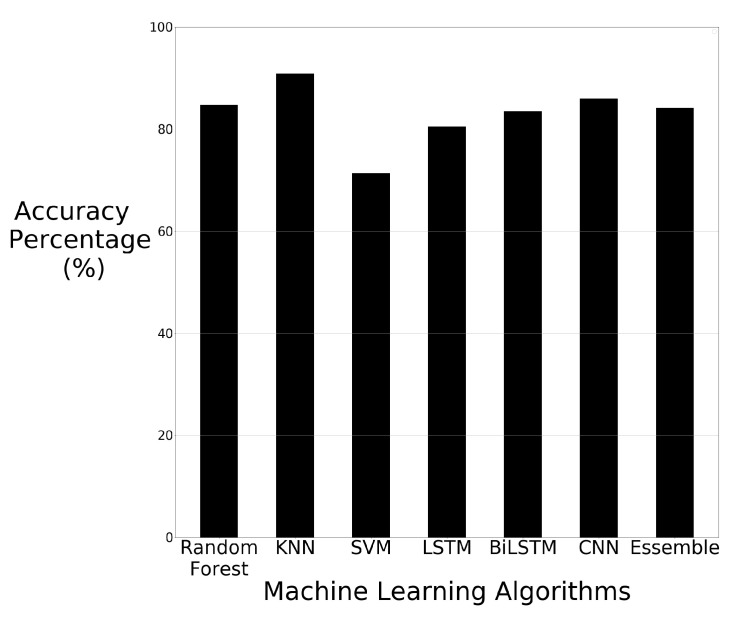
Machine learning results using raw image data.

The accuracy results of each machine learning algorithm using just the raw images are visually displayed in bar chart format in Figure 6.

The results of the machine learning algorithms show that KNN has superior performance using the raw image data. KNN has an accuracy of 90.85%. Although KNN performs the best, all the algorithms can classify the majority of the images correctly. These results show that the machine learning algorithms applied to the image data are able to identify the differences in the image data, with KNN being able to distinguish the differences at a higher rate than the other algorithms. The other algorithms were still able to recognise differences in the images, however, CNN was the second-best performer with 85.97% accuracy. SVM is the weakest performer out of all algorithms but still achieved 71.34% accuracy.

Table 2 shows the accuracy and the average precision, recall and F1-score of the machine learning algorithms using the raw images.

#### 4.1.2. Principal Component Analysis

**Figure 7:**
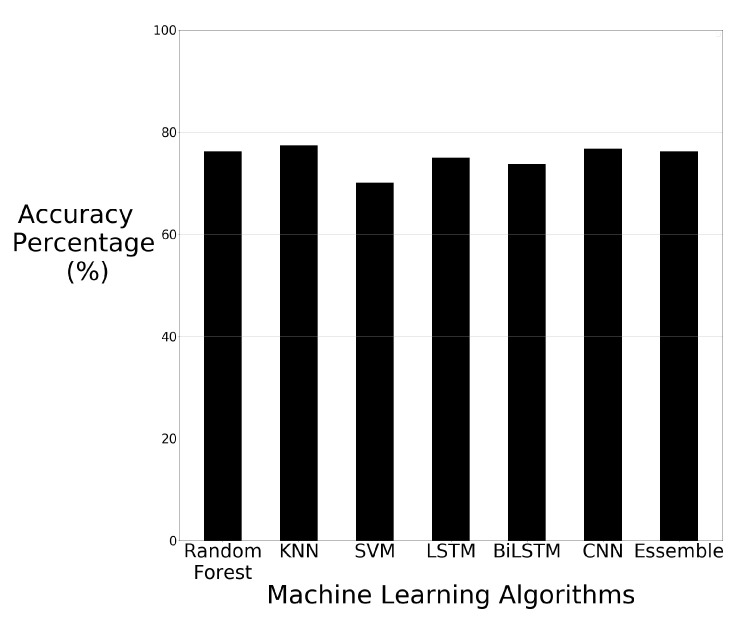
Machine learning results using raw image data with PCA.

The accuracy results of each machine learning algorithm using PCA are visually displayed in bar chart format in Figure 7.

The results of the PCA described a drop in comparison to using the raw image data. KNN maintained its status as being the highest performer of the machine learning algorithms but it dropped by 13.42% in accuracy from 90.85% when using raw data to 77.43% when using PCA. SVM had the smallest drop in performance compared to the other algorithms when using PCA with a drop of 1.22%, from 71.34% down to 70.12%. These results show that using PCA decreases the accuracy compared to the results of using raw data. The deep learning algorithms were still ultimately able to differentiate between human motions using PCA despite the drop in accuracy.

Table 2 shows the accuracy and the average precision, recall and F1-score of the machine learning algorithms and Table 3 shows the average precision, recall and F1-score of the machine learning algorithms using PCA for the image data.

### 4.2. Data Augmentation

#### 4.2.1. Raw Images

**Figure 8:**
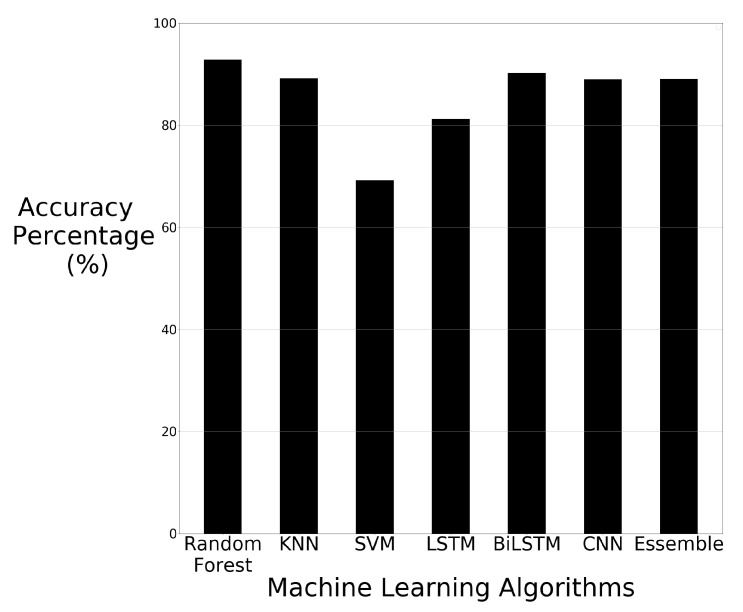
Machine learning results using data augmentation.

The accuracy results of the machine learning algorithms using data augmentation is visually displayed in bar chart format in Figure 8.

The results of using data augmentation have shown that random forest increased in accuracy with the benefits of having more training data in comparison to only using raw images with no data augmentation. The accuracy of random forest increased by 8.1%, from 84.75% to 92.85%. KNN and SVM showed decreased accuracy scores with the added training data. The accuracy of KNN dropped by 1.67%, from 90.85% to 89.18%. The drop in accuracy with SVM was 2.16%, from 71.34% down to 69.18%. SVM still remains the lowest performer out of all the machine learning algorithms, whereas random forest outperformed KNN and was the only algorithm that benefited from the larger dataset. The BiLSTM algorithm showed increased accuracy by 6.67%, from 83.53% to 90.20%, making it the second best-performing algorithm. The CNN algorithm had an accuracy of 88.97% when using the data augmentation dataset. It had an accuracy increase of 3.00% compared to using the raw image dataset. The LSTM did improve slightly when using the data augmentation dataset with an increase of 0.74%, from 80.48% to 81.22%. Table 4 shows the accuracy and the average Precision, recall and F1-score of the machine learning algorithms using data augmentation techniques.

#### 4.2.2. Principal Component Analysis

**Figure 9:**
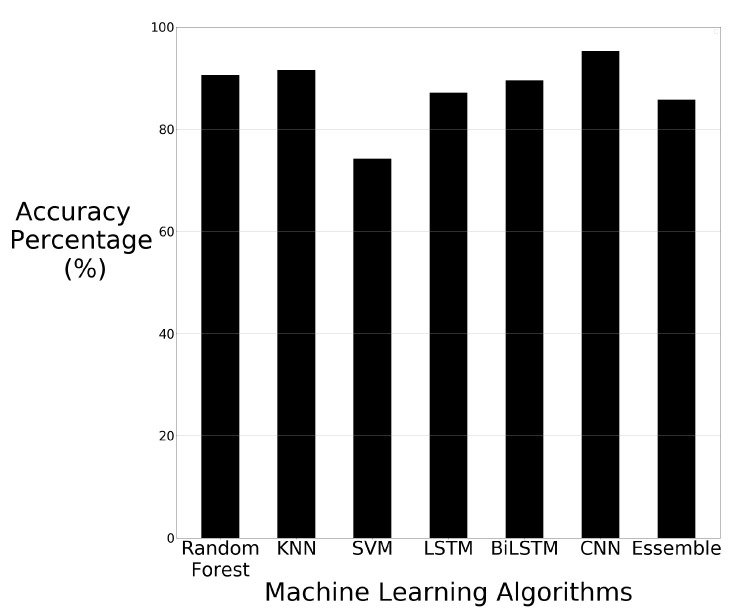
Machine learning results using data augmentation with PCA.

The accuracy results of the machine learning algorithms using data augmentation with PCA are displayed in Figure 9.

The machine learning results using PCA and data augmentation have shown that KNN, SVM, LTSM and CNN improved when using PCA and data augmentation in comparison to just using data augmentation. The KNN results increased from 89.18% to 91.63% compared to using only data augmentation techniques. SVM results increased from 69.18% to 74.28% compared to using only data augmentation techniques. LSTM showed an increased accuracy result from 81.22% up to 87.14% and CNN showed increased accuracy from 88.97% up to 95.30%. The result from CNN is the highest achieved result in all experiments undertaken throughout this study.

When the KNN, SVM, LTSM and CNN algorithms were applied to the PCA dataset without data augmentation, the results decreased in performance. These results show that for KNN, SVM, LTSM and CNN, the additional training data help to improve the performance. The random forest and BiLTSM algorithms showed a decrease in performance when using PCA and data augmentation in comparison to only using data augmentation. The accuracy only fell by 2.24%, from 92.85% down to 90.61%, for random forest and BiLTSM fell from 90.20% to 89.59%. This drop is significantly less severe compared to the drop observed in the results of random forest and CNN when using raw images to then using the raw images with PCA. Random forest dropped in accuracy by 8.54% and BiLTSM dropped in accuracy by 9.75%. This shows that PCA performs better in most of the machine learning algorithms when the amount of training data is increased, with the exception being the random forest and CNN algorithms. Table 5 shows the accuracy and the average Precision, recall and F1-score of the machine learning algorithms using data augmentation techniques paired with PCA.

### 4.3. Ablation Studies

**Figure 10:**
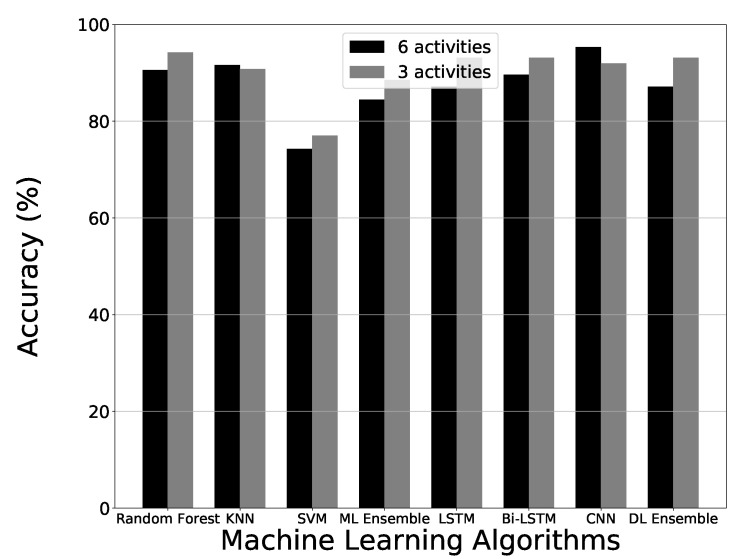
Ablation study results using data augmentation paired with PCA.

This section details ablation. Ablation is the process of removing activities within the dataset and an analysis of how the reduced number of classifications compares to results achieved using the complete dataset. For this section, the best performing dataset when using data augmentation and PCA was reduced from six activities down to three. The three activities used were walking, sitting and standing. Figure 10 shows the accuracy of the comparison of each algorithm between three and six activities. Most of the algorithms achieved higher results when using fewer classifications, however, KNN and CNN algorithms performed worse with three activities. The difference with KNN was negligible and the difference in performance with CNN was likely due to the reduced amount of training data. In experiments using six activities, it was observed that CNN performs better with more training data.

## 5. Comparison to State-of-the-Art Approaches

The results of our proposed approach can be directly compared to the results of [18], as this work used the same dataset used in the experiments of this study. The authors of [18] used SVM and KNN algorithms and these algorithms achieved an accuracy score of 78.25% and 77.15%, respectively. SVM, in the results presented in this paper, achieved the highest result of 74.28%, which was lower than the work shown in [18]. However, we were able to improve the KNN results in our work. In our experimentation, KNN achieved its highest result of 91.63% when using data augmentation paired with PCA. The data processing techniques used in this study have allowed for the KNN algorithm to improve the accuracy results by nearly 15%. The work conducted in this study even expanded on the improvements in accuracy using the dataset with the use of CNN. The accuracy score of 95.30% achieved when using CNN with data augmentation and PCA was the best accuracy score achieved in this work, showing an accuracy score increase of around 17% from the best accuracy score achieved by [18] when using the KNN algorithm.

## 6. Conclusions

This paper presented results obtained when machine and deep learning techniques were applied to a radar dataset that was previously generated containing radar micro-Doppler spectrograms for various human activities. The research work has shown that using PCA paired with augmentation of the available data achieved the best results of 95.30% using the deep learning algorithms, namely CNN. These results have outperformed the state-of-the-art approaches. The results also highlighted how PCA can achieve better results when the training data are expanded using data augmentation. Without data augmentation, it was found that PCA had a negative effect on the results. Although the highest machine learning result was obtained using random forest without PCA, the rest of the machine learning algorithms displayed higher results when PCA was used with data augmentation. The results of this paper have shown that AI can identify the different activities of daily living that are detected by radar sensing by performing image classification on acquired micro-Doppler signatures. Future work of this research can seek to implement systems that make use of the data processing and deep learning techniques presented in the findings of this paper to improve the lives of elderly people.

## Figures and Tables

**Table 1:** Number of spectrograms per motion in the dataset.

Algorithm	Number of Samples
Walking	286
Sitting Down	289
Standing Up	287
Picking Up an Object	287
Drinking Water	286
Falling	198
Total	1633

**Table 2:** Machine learning results using raw image data.

Algorithm	Accuracy	Precision	Recall	F1-Score
Random Forest	84.75%	0.86	0.85	0.85
KNN	90.85%	0.91	0.91	0.91
SVM	71.34%	0.74	0.71	0.71
LSTM	80.48%	0.83	0.80	0.80
BiLSTM	83.53%	0.87	0.84	0.84
CNN	85.97 %	0.86	0.86	0.86

**Table 3:** Machine learning results using raw image data with PCA.

Algorithm	Accuracy	Precision	Recall	F1-Score
Random Forest + PCA	76.21%	0.78	0.76	0.76
KNN + PCA	77.43%	0.78	0.77	0.77
SVM + PCA	70.12 %	0.71	0.70	0.70
LSTM + PCA	75.00%	0.76	0.75	0.75
BiLSTM + PCA	73.78%	0.74	0.74	0.74
CNN + PCA	76.82%	0.78	0.77	0.77

**Table 4:** Machine learning results using data augmentation.

Algorithm	Accuracy	Precision	Recall	F1-Score
Random Forest	92.85%	0.93	0.93	0.93
KNN	89.18%	0.89	0.89	0.89
SVM	69.18%	0.70	0.69	0.69
LSTM	81.22%	0.83	0.81	0.81
BiLSTM	90.20%	0.90	0.90	0.90
CNN	88.97%	0.91	0.89	0.89

**Table 5:** Machine learning results using data augmentation with PCA.

Algorithm	Accuracy	Precision	Recall	F1-Score
Random Forest + PCA	90.61%	0.91	0.91	0.91
KNN + PCA	91.63 %	0.92	0.92	0.92
SVM + PCA	74.28 %	0.75	0.74	0.74
LSTM + PCA	87.14%	0.88	0.87	0.87
BiLSTM + PCA	89.59%	0.90	0.90	0.90
CNN + PCA	95.30%	0.95	0.95	0.95

## Data Availability

The dataset can be found at http://researchdata.gla.ac.uk/848/, accessed on 20 April 2021.
